# Predicting Factors Affecting Survival Rate in Patients Undergoing On‐Pump Coronary Artery Bypass Graft Surgery Using Machine Learning Methods: A Systematic Review

**DOI:** 10.1002/hsr2.70336

**Published:** 2025-01-22

**Authors:** Alireza Jafarkhani, Behzad Imani, Soheila Saeedi, Amir Shams

**Affiliations:** ^1^ Department of Operating Room, School of Paramedicine Hamadan University of Medical Sciences Hamadan Iran; ^2^ Department of Health Information Technology, School of Allied Medical Sciences Hamadan University of Medical Sciences Hamadan Iran; ^3^ Department of Cardiac Surgery, School of Medicine Hamadan University of Medical Sciences Hamadan Iran

**Keywords:** coronary artery bypass, machine learning, survival rate, systematic review

## Abstract

**Background and Aim:**

Coronary artery bypass grafting (CABG) is a key treatment for coronary artery disease, but accurately predicting patient survival after the procedure presents significant challenges. This study aimed to systematically review articles using machine learning techniques to predict patient survival rates and identify factors affecting these rates after CABG surgery.

**Methods:**

From January 1, 2015, to January 20, 2024, a comprehensive literature search was conducted across PubMed, Scopus, IEEE Xplore, and Web of Science. The review adhered to the Preferred Reporting Items for Systematic Reviews and Meta‐Analyses (PRISMA) guidelines. Inclusion criteria included studies that evaluated survival rates and predictors associated with CABG patients during the specified period.

**Results:**

After eliminating duplicates, a total of 1330 articles were identified. Following a systematic screening, 24 studies met the inclusion criteria. Our findings revealed 43 distinct factors influencing survival rates in patients undergoing CABG. Notably, five factors—age, ejection fraction, diabetes mellitus, a history of cerebrovascular disease or accidents, and renal function—were consistently identified across multiple studies as significant predictors of postsurgical survival.

**Conclusion:**

This systematic review identifies key factors influencing survival rates after CABG surgery and highlights the role of machine learning in improving predictive accuracy. By identifying high‐risk patients through these key factors, our findings offer practical insights for healthcare providers, enhancing patient management and customizing therapeutic strategies after CABG. This study significantly enhances existing literature by combining machine learning techniques with clinical factors, thereby improving the understanding of patient outcomes in CABG surgery.

## 1 Introduction

Cardiovascular diseases (CVDs) are responsible for the most significant number of deaths worldwide, particularly in low and middle‐income countries. Approximately 17 million individuals lose their lives each year due to these diseases, and this number is predicted to rise to approximately 24 million by 2030. In Iran, it is expected that the prevalence of CVDs will double by 2025 compared to the previous two decades [1, 2, 3]. Family history, diabetes mellitus, smoking, and hypertension are among the risk factors related to CVDs [4]. Coronary artery disease (CAD) is one of the most common CVDs. Therapeutic measures for this disease include lifestyle changes, drug therapy, and surgery [5, 6].

Coronary artery bypass surgery (CABG) is a successful treatment for CADs. Despite being expensive, the risks of the surgery are very low. Even with the rise of percutaneous coronary intervention (PCI), CABG remains the most frequently performed heart surgery [7, 8, 9].

One of the essential considerations in surgical decision‐making is identifying patients who will benefit from surgery after tissue damage. While overall survival is an important criterion, it is complex to assess the overall benefit of surgery [10, 11]. Due to the advancements in surgical care and techniques and the decrease in surgical mortality rates, there is a need to focus more on improving the survival rate of patients after surgery. Accurately predicting patient survival rates can help healthcare professionals determine the most effective strategies for postdischarge care [12, 13]. The survival rate of patients after CABG surgery has been reported differently in studies [14, 15]. For example, according to one study conducted in 2021, the 1‐year survival rate of patients after surgery was about 93%, and the 5‐year survival rate was about 83% [15]. Considering that CABG surgery is one of the most common and costly surgeries performed today, it is essential to develop an interpretable tool that shows the relationship between physiological, operative, and postoperative variables and patients' long‐term survival rates [16].

AI has significantly contributed to computer science and related fields in recent decades. Machine learning is one of the branches of artificial intelligence to analyze large amounts of data [17, 18, 19]. Machine learning involves using statistical algorithms and models by computer systems to perform a task without explicit instructions. These systems learn from previously available data and use mathematical models to predict future outcomes [20, 21]. Recent studies have shown that machine learning methods can be used to indicate the survival rate of patients after heart surgeries [22, 23], as well as for various types of cancer, including breast [24], cervical [25], and colorectal cancers [26]. Furthermore, machine learning methods have also been employed to predict the survival rate of patients suffering from Covid‐19 [27].

Various studies have investigated the survival rate of patients after CABG surgery and the factors affecting it, given the crucial role of the survival rate of patients and the potential of machine learning algorithms in predicting the future. There are differences in the survival rate of patients and the factors affecting it in various studies that utilized machine learning methods. Therefore, we decided to conduct a systematic review study to investigate the survival rate of patients after surgery along with the influencing factors.

## 2 Materials and Methods

The present study followed the Preferred Reporting Items for Systematic Reviews and Meta‐Analyses (PRISMA) proposed by Moher et al. [28] To ensure relevant studies were included.

### 2.1 Search Strategy

This review searched Web of Science, Scopus, IEEE Library, and PubMed to identify relevant studies published from January 1, 2015 until January 20, 2024. The search strategy included combined keywords and mesh terms related to machine learning, CABG surgery, and survival rate. A complete list of keywords is given in Table 1.

**Table 1:** Keywords related to searching databases.

Keywords
((“Coronary Artery Bypass”[Mesh]) OR (“Artery Bypass, Coronary”[Title/Abstract]) OR (“Artery Bypasses, Coronary”[Title/Abstract]) OR (“Bypasses, Coronary Artery”[Title/Abstract]) OR (“Coronary Artery Bypasses”[Title/Abstract]) OR (“Coronary Artery Bypass Surgery”[Title/Abstract]) OR (“Bypass, Coronary Artery”[Title/Abstract]) OR (“Aortocoronary Bypass”[Title/Abstract]) OR (“Aortocoronary Bypasses”[Title/Abstract]) OR (“Bypass, Aortocoronary”[Title/Abstract]) OR (“Bypasses, Aortocoronary”[Title/Abstract]) OR (“Bypass Surgery, Coronary Artery”[Title/Abstract]) OR (“Coronary Artery Bypass Grafting”[Title/Abstract]) OR (“Open Heart Surgery”[Title/Abstract]) OR (“Open Cardiac Surgery”[Title/Abstract])) AND ((“Machine learning”[Title/Abstract]) OR (“Machine Learning”[Mesh]) OR (“Deep Learning”[Mesh]) OR (“Deep Learning”[Title/Abstract]) OR (“Data Mining”[Title/Abstract]) OR (“Neural Network”[Title/Abstract]) OR (“Genetic Algorithms”[Title/Abstract]) OR (“Support Vector Machine”[Title/Abstract]) OR (“Support Vector Machine”[Mesh]) OR (“Support Vector Network”[Title/Abstract]) OR (“Support Vector Networks”[Title/Abstract]) OR (“Supervised Machine Learning”[Mesh]) OR (“Unsupervised Machine Learning”[Mesh]) OR (“Supervised Machine Learning”[Title/Abstract]) OR (“Unsupervised Machine Learning”[Title/Abstract]) OR (“Random Forest”[Title/Abstract]) OR (“Random Forest”[Mesh]) OR (“Random Forests”[Title/Abstract]) OR (“Bayes Theorem”[Mesh]) OR (“Bayesian Network”[Title/Abstract]) OR (“Artificial Neural Network”[Title/Abstract]) OR (“Clustering”[Title/Abstract]) OR (“Decision Tree”[Title/Abstract]) OR (“Decision Trees”[Mesh]) OR (“Regression”[Title/Abstract]) OR (“Bayesian”[Title/Abstract]) OR (“Naive Bayes”[Title/Abstract]) OR (“K‐Nearest Neighbors”[Title/Abstract]) OR (“K‐Nearest Neighbor”[Title/Abstract]) OR (“Genetic Algorithms”[Title/Abstract]) OR (“Gradient Boosting”[Title/Abstract])) AND ((survival [Title/Abstract]) OR (survivor[Title/Abstract]) OR (surviving[Title/Abstract]) OR (survive[Title/Abstract]))

### 2.2 Selection Criteria

Articles were included if they met the following criteria:

1. Studies that precisely predict survival rate (nor mortality rate) and its related factors after CABG, not, for example, all cardio or cardiovascular surgery, open heart surgery, or CABG combined with valve surgery.
2. Studies predicting survival rate and its related factors after CABG using the on‐pump technique, excluding surgeries in which the patient did not receive on‐pump bypass or in which survival rate was compared between on‐pump and off‐pump surgeries.
3. Studies in which only the conventional CABG method was used, excluding those performed with robotic, hybrid, or minimally invasive methods.
4. Studies that did not include children in their study population.

If a study had any of the following criteria, it was excluded from the study:

1. The article's title, abstract, or full text was irrelevant to our aim.
2. Studies that were part of different chapters of books, letters to the editor, short summaries, case reports, or part of a structured review method with/without meta‐analysis.
3. Studies whose full text was not available.
4. Studies that were published in a language other than English.
5. Studies that were only to develop and test various machine learning models to predict the survival rate and related factors.

### 2.3 Study Selection

After a thorough and organized search, all the studies found were inserted into EndNote X9 citation management software. After removing duplicate entries, two authors (A.J. and S.S.) independently screened the titles and abstracts of all the studies based on the study criteria. Once the relevant articles that met the criteria were identified, the full texts of those articles were reviewed by two researchers. A final decision was made regarding the inclusion of the relevant studies. Any discrepancies or inconsistencies were resolved through discussion and coordination with the study's supervisor, B.I. The full texts of the reviewed articles that did not meet the inclusion criteria were excluded from the study, and the reasons for exclusion were also checked by the research team. In the final step, the necessary data was extracted from the articles in this systematic review and entered into Excel software.

### 2.4 Data Extraction

Two researchers extracted the data using a form designed in Excel. Any discrepancies were resolved through discussions between the researchers and the study supervisor. The extracted data include the study, first author name, year of publication, the country, the name of the journal, source of data, the sample size of the study, source of data, the machine learning methods used, survival rate, predictive factors of survival rate, whether or not there were any limitations, data types, whether the study was retrospective or prospective and target group.

### 2.5 Risk of Bias

The risk of bias in the included articles was evaluated by two independent reviewers using the Cochrane Collaboration Risk of Bias Tool, as recommended by Narukab [29] for machine learning‐related studies. The methodological quality of the articles was assessed based on the following domains: [1] Data collection, [2] Study response, [3] Outcome measurement, and [4] Statistical analysis and reporting. Each study was categorized as high risk, unclear risk, or low risk of bias.

### 2.6 Data Analysis

No meta‐analysis was performed due to heterogeneous methods and reporting in the included studies. Selected studies were reported via narrative synthesis.

## 3 Results

### 3.1 Study Selection

**Figure 1:**
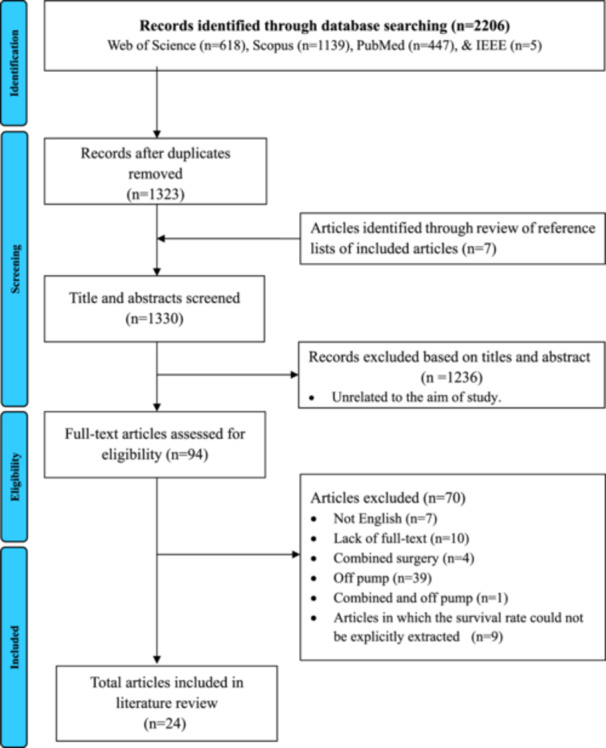
Flow diagram of the literature search and study selection. This figure illustrates the stages of screening studies, from identification to inclusion. Given the study's objectives and the significance of inclusion and exclusion criteria, 24 studies were ultimately included in the final analysis out of the initial 2206 studies retrieved in the first stage.

Searching in four databases, PubMed, Scopus, IEEE Xplore, and Web of Science, from January 1, 2015, until January 20, 2024, led to identifying 2206 articles. Out of this number of retrieved articles, 883 duplicate articles were identified and removed with the help of EndNnote software and reviewed by researchers. After screening the title and abstract of the articles based on the defined inclusion and exclusion criteria, 94 articles remained. After examining the full text of these articles, 24 articles were finally included in the analysis (Figure 1).

### 3.2 General Characteristics of the Included Studies

**Figure 2:**
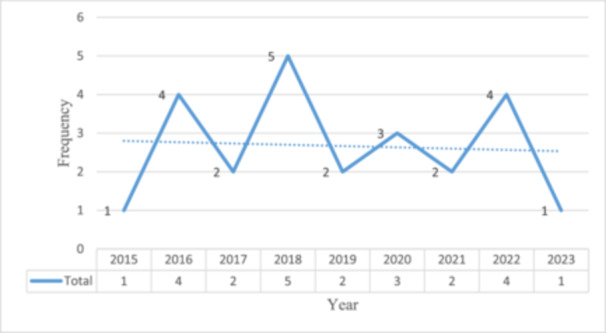
Distribution of published studies by year. The figure represents the number of articles published from 2015 to 2023. It clearly shows that the highest number of articles published in our field is in 2018.

Table 2 gives the general characteristics of the articles included in this systematic review. The articles were published between 2015 and 2023. In 2018, the largest number of articles (*n* = 5) were published; in 2016 and 2022, four were published (Figure 2).

**Table 2:** General characteristics of studies included in the systematic review.

First author	Year of publication	Country	Source of data	Sample size	Methods	Survival rate	Data types	Study design	Target group
Salmasi [30]	2023	UK	Database at a single cardiothoracic institution Hospital records	1957	Cox regression Logistic regression Linear regression Kaplan−Meier	10‐year survival between South‐Asian: 83.2% 10‐year survival between non‐Asian: 85.9%	Clinical data	Retrospective	Isolated CABG patients
Jonsson [31]	2022	Sweden	SWEDEHEART National healthcare registries in Sweden Swedish Heart Surgery Registry, which is a part of the SWEDEHEART registry National Cause of Death Registry	36,898	Cox regression Logistic regression Kaplan−Meier	Overall survival rate in stroke: 49.2 Overall survival in non‐stroke: 80.8	Clinical data	Prospective	A patient that has a stroke after CABG
Jegaden [32]	2022	UAE	Surgical registry of a department	1432	Cox regression Logistic regression Kaplan−Meier	Unmatched Vein: 5‐year: 87 ± 3, 10‐year: 70 ± 4, 15‐year: 51 ± 5, 20‐year: 33 ± 6 Unmatched GEA: 5‐year: 91 ± 2, 10‐year: 79 ± 3, 15‐year: 65 ± 4, 20‐year: 48 ± 4 Matched vein: 5‐year: 87 ± 4, 10‐year: 71 ± 5, 15‐year: 53 ± 6, 20‐year: 36 ± 7 Matched GEA: 5‐year: 91 ± 3, 10‐year: 78 ± 5, 15‐year: 60 ± 6, 20‐year: 41 ± 7	Clinical data	Retrospective	Isolated CABG patients using BITA
Hendriks [33]	2022	The Netherlands	Cardiothoracic operation registry Hospital database Dutch Municipal Database	5672	Cox regression Kaplan−Meier Machine learning‐based clustering	30 days and 5‐year survival were 98% and 88% in the mild hypothermia group, whereas it amounted to 93% (< 80% in the severe hypothermia 30°C). Normothermia (35°C–37°C) showed the lowest survival after 30 days and 5 years, amounting to 93% and 72%	Clinical data	Retrospective	Isolated CABG patients with CPB
Omer [34]	2022	USA	Veterans Affairs Surgical Quality Improvement Program data	61,477	Cox regression Kaplan−Meier Multivariable hierarchical regression	Overall survival at 10 years was 61.5%, 41.1%, and 36.7% for LVEF 35%, 25%−34%, and < 25%	Clinical data	Retrospective	Isolated CABG patients
Han [35]	2021	USA	Society of Thoracic Surgeons (STS) Adult Cardiac Surgery Database Electronic medical record system	7652	Cox regression Logistic regression Kaplan−Meier	survival rates at 1 year were 87.8%, 84.7%, 83.9%, and 88.9% in surgeon experience quartiles 1, 2, 3, and 4 5‐year survival rates were 77.8%, 67.0%, 66.1%, and 77.9% 10 year survival rates were 65.7%, 52.6%, 59.5%, and 75.4%	Clinical data	Retrospective	Isolated CABG patients
Sattartabar [36]	2021	Iran	The coronary angiography data bank	24,328	Cox regression Logistic regression Kaplan–Meier	Men under 50 had the best survival (93.6%) Worst survival was observed in female patients older than 55 (83.4%) In general, patients had 85.9% overall survival and 74.1%	Clinical data	Retrospective	Isolated CABG patients
Parker [37]	2020	Lebanon	Northern New England Cardiovascular Disease Study Group Cardiac Surgery Registry	1560	Cox regression Kaplan−Meier	6‐year survival rate: 86%	Clinical data	Prospective	Isolated CABG patients
Zarei [38]	2020	Iran	Patients participated in the Tehran Heart Center‐Coronary Outcome Measurement (THC‐COM) cohort study	404	Cox regression Kaplan−Meier	The mean survival rate for CAD patients with 25 (OH)D < 20 was 73.6 months CAD patients with 25(OH)D between 20 and 30 was 72.6 months Those with 25(OH)D > 30 was 73.9 months	Clinical data	Prospective	Isolated CABG patients
Gallo [39]	2020	USA	A single‐center adult cardiac surgery database	2713	Logistic regression Kaplan−Meier	The survival at 1, 3, and 5 years for redo‐CABG was 93.5%, 90%, and 85%, respectively, and 95.5%, 94.5%, and 93% for first‐time CABG	Clinical data	Retrospective	2581 patients underwent first‐time CABG procedures, while 132 underwent isolated redo‐CABG
Robich [40]	2019	USA	Northern New England Cardiovascular Disease Study Group (NNECDSG) Cardiac Surgery Registry National Death Index Social Security Administration Master Death File	6415	Cox regression Logistic regression Kaplan−Meier	Survival rates by increasing HbA1c group were 93%, 92%, 90%, and 88% at 3 years and 89%, 86%, 83%, and 79% at 5 years	Clinical data	Retrospective	Patient undergoing CABG (diabetic and nondiabetic)
Wang [41]	2019	China	Medical records and the database	1144	Logistic regression Cox regression	Long‐term survival in group A was higher than in group B (96.2% vs. 93.1%)	Clinical data	Retrospective	Patients with preoperative eGFR of more than 60 mL/min/1.73 m^2^ receiving first isolated CCABG surgery
Karim [42]	2018	Australia	Australian and New Zealand Society of Cardiac and Thoracic Surgeons registry National Death Index (NDI) Database	46,573	Cox regression Kaplan−Meier	90 days: 98.3 1 year: 97.2 2 years: 95.6 3 years: 93.9	Clinical data	Retrospective	Isolated CABG patients
Luthra [43]	2018	UK	New Cross Hospital database National UK database National Institute of Cardiac Outcomes Research (NICOR) database	598	Cox regression Kaplan−Meier	30‐day mortality was 0.6% in both groups Venous versus arterial 99.2% versus 99.2%; at 1 year; 85.2% versus 88.8%; at 5 years and 69.2% versus 88.8%; at 7 years	Clinical data	Retrospective	Isolated CABG patients
Cheng [44]	2018	China	Data collected from patients	600	Cox regression Logistic regression Kaplan−Meier	Survival for patients who had in‐hospital VT/VF was lower than that of the non‐VT/VF group (89.9% vs. 97.6%)	Clinical data	Prospective	Patients with impaired left ventricular function undergoing CABG
Beller [45]	2018	USA	Institutional Society of Thoracic Surgeon database Institutional Clinical Data Repository (CDR)	1272	Logistic regression Cox regression Kaplan−Meier	Median long‐term survival was decreased in the preoperative positive troponin group (12.5 vs. 13.6 years)	Clinical data	Retrospective	Isolated CABG patients who had preoperative troponin measurements
Malaisrie [46]	2018	USA	STS Adult Cardiac Surgery Database	347,977	Cox regression Logistic regression Kaplan−Meier	At 5 years, in the preoperative atrial fibrillation versus no atrial fibrillation groups stratified by CHA2DS2‐VASc scores were 74.8% versus 86.3%, 56.5% versus 73.2%, and 41.2% versus 57.2%	Clinical data	Retrospective	Isolated CABG patients
Benedetto [47]	2017	UK	National Institute for Cardiovascular Outcomes Research (NICOR) NACSA registry Institutional database General Register Office	12,244	Cox regression Logistic regression	The probability of survival at 5, 10, and 15 years in the RA group was 93.8% ± 0.6%, 84.2% ± 0.9%, and 69.4% ± 1.9% compared to 87.7% ± 0.3%, 70.9% ± 0.5%, and 51.4% ± 0.08% in the unmatched SV group and 90.9% ± 0.7%, 79.5% ± 1.1%, and 63.9% ± 1.6% in the PS‐matched SV group	Clinical data	Retrospective	Patient undergoing CABG (obese and not obese)
Luthraa [48]	2017	UK	New Cross Hospital database National UK database National Institute of Cardiac Outcomes Research (NICOR) database	1226	Cox regression Logistic regression Kaplan−Meier	Arterial versus venous: 98.2% versus 96.3%, at 1 year; 82.4% versus 62.2%, at 10 years	Clinical data	Retrospective	Isolated CABG patients
Najafi [49]	2016	Iran	Databases were completed from THC surgery Telephone interviews Online registration of deaths	566	Cox regression Kaplan−Meier	Five‐year overall survival was 91.8%; 86.6% in opium consumers and 92.7% in non‐opium consumers	Clinical data	Prospective	Isolated CABG patients
Efird [50]	2016	USA	STS Adult Cardiac Surgery Database Electronic medical record (EMR) The National Death Index (NDI)	4801	Cox regression Kaplan−Meier	Five‐year survival rates for non‐COPD patients with and without PLOS were 54% and 88% The 5‐year survival rates for COPD patients with and without PLOS were 30% and 76%	Clinical data	Retrospective	COPD and non‐COPD patients after CABG
Pang [51]	2016	Singapore	Hospital records National Registry of Deaths	5720	Cox regression Logistic regression Kaplan−Meier	In the unmatched cohort, overall 20‐year survival rates were 30.9% ± 1.6% in people with diabetes and 49.2% ± 1.0% in non‐diabetics In the propensity‐matched group, overall 20‐year survival rates were 35.4% ± 2.5% in people with diabetes and 48.9% ± 2.9% in non‐diabetics	Clinical data	Retrospective	CABG patients
Farhadi [52]	2016	Iran	Medical files Telephone contact Personal visits	269	Cox regression Kaplan−Meier	One month, 6 months, 1 year, and 3 years survival of CABG patients was 96.7%, 95.3%, 94.7%, and 89.8%, respectively For the pharmaceutical medication patients were 97%, 92.5%, 86.3%, and 83.4% Survival term in MFU and CABG patients was 52.80 ± 1.64 and 57.15 ± 1.23 months, respectively	Clinical data	Retrospective	The 3VD patients underwent coronary artery angiography and had EF < 30%
Singh [53]	2015	USA	Medical records Society of Thoracic Surgeons (STS) National Cardiac Database Cardiac catheterization laboratory (Apollo system) Social Security Death Index	1141	Cox regression Logistic regression Kaplan−Meier	The overall 30‐day survival: 94.3	Clinical data	Retrospective	Isolated CABG in octogenarian patients

**Figure 3:**
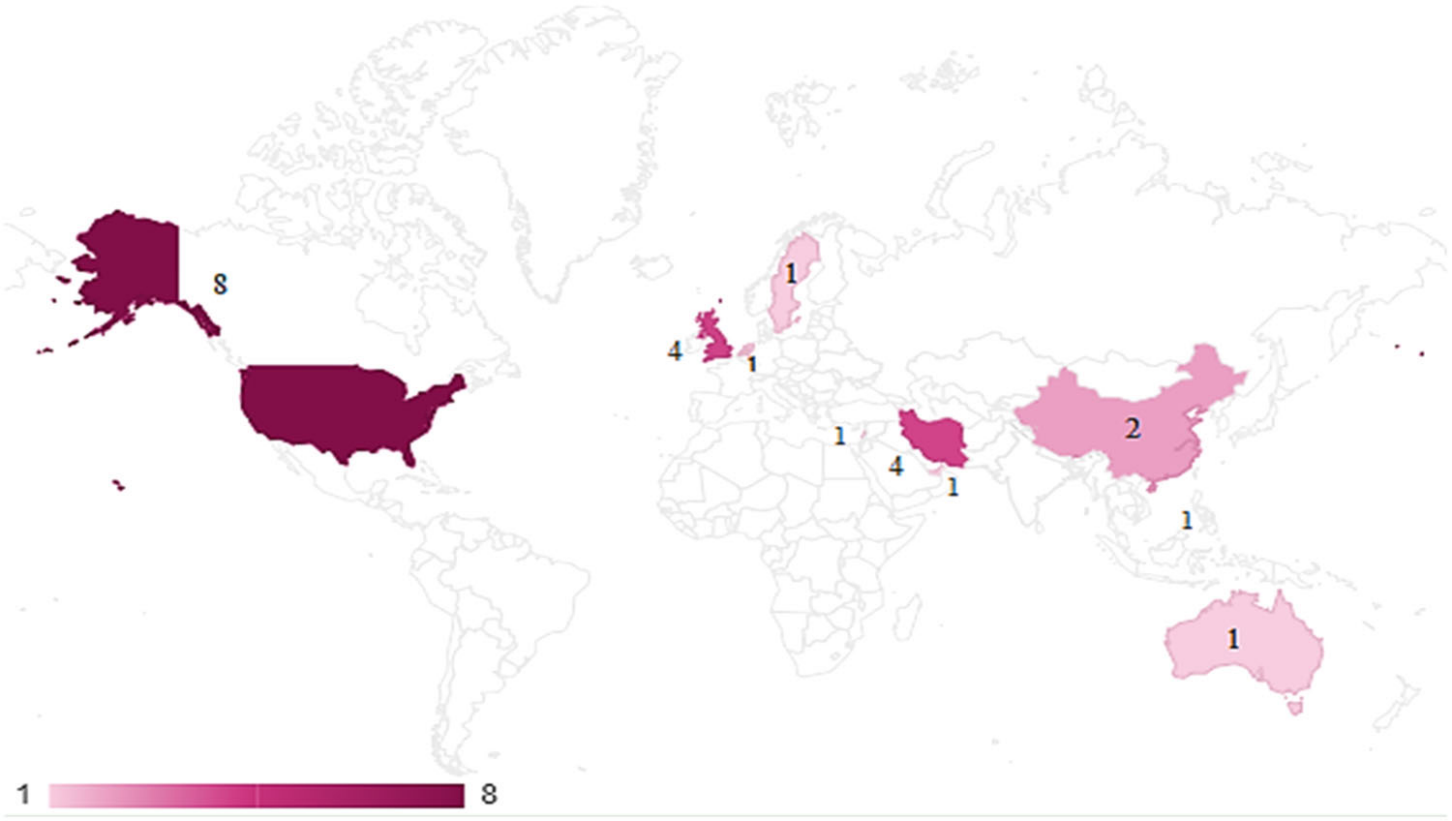
Distribution of published studies by country. The figure illustrates the number of articles published on our topic in various countries. It appears that patient survival following on‐pump CABG surgery is particularly popular among authors in the United States.

Figure 3 shows the distribution of published studies by country. The USA conducted the most studies, eight (33.33%), and Iran and the UK were in the next rank with four studies each.

### 3.3 Distribution of Studies Based on Journals

**Figure 4:**
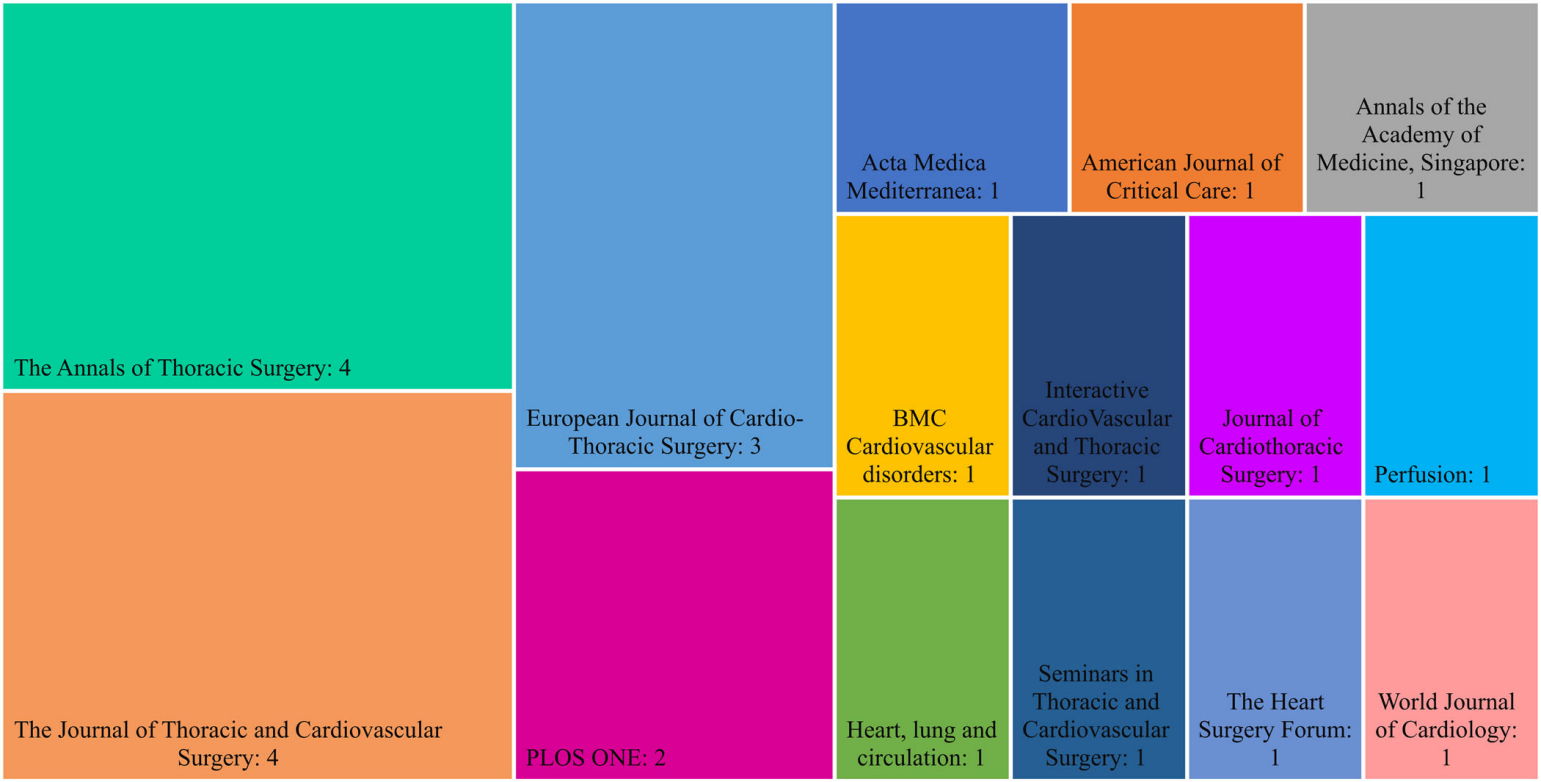
Distribution of published studies by the journal. This figure displays the published articles reviewed in our study across different journals. The two journals in the first column on the left appear to be the most popular choices for accepting these articles, each publishing eight articles authored by the researchers.

The distribution of studies based on the journals that published articles related to predicting the survival rate of patients undergoing CABG is shown in Figure 4. The journals “*The Journal of Thoracic and Cardiovascular Surgery*” and “*The Annals of Thoracic Surgery*” with the publication of four articles, and the “*European Journal of Cardio‐Thoracic Surgery*,” with the publication of three articles, were the journals that had published the largest number of articles in this field.

### 3.4 Source of Data and Sample Size

The data used in the studies were collected retrospectively (*n* = 19, 79.2%) and prospectively (*n* = 5, 20.8%). The data sources in the reviewed articles included national or local registries and databases, medical records, telephone contact, and personal visits. The Society of Thoracic Surgeons (STS) Adult Cardiac Surgery Database was used more than other registries.

The smallest sample size used to predict the survival rate of CABG patients was 269, and the largest was 347,977. The sample median in the included articles was 2335.

### 3.5 The Distribution of Included Papers Based on Applied Analysis Methods

**Figure 5:**
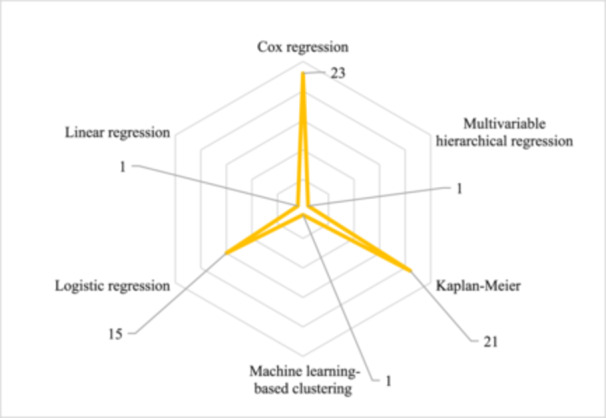
Distribution of published studies based on applied analysis methods. This figure illustrates the distribution of machine learning methods utilized in studies. Cox regression is the most commonly used machine learning technique for data analysis in the studies conducted.

The review of the articles included in this systematic review revealed that different methods were used to analyze the data and predict the survival rate of patients undergoing CABG. The three methods used for data analysis were Cox regression, Kaplan−Meier, and Logistic regression, which were used 23, 21, and 15 times, respectively. Other methods were also used once each (Figure 5).

### 3.6 Factors Affecting Survival Rate in CABG Patients

Factors affecting mortality and survival rates of patients who underwent CABG were extracted from the studies included in this systematic review (Table 3). These elements included 43 factors. Among these factors, age, ejection fraction, diabetes, cerebrovascular disease/accident, and renal function were the most repeated in the studies.

**Table 3:** Factors affecting survival rate.

	Predictors	Studies
#	Peripheral vascular disease	[42], [31]
1	Congestive heart failure	[42]
2	Hypertension	[42], [36]
3	Diabetes	[42] [49], [32], [36]
4	Steroid use	[42]
5	Cerebrovascular disease/accident	[42] [31], [49]
6	Age	[42] [31], [49], [32], [33], [51], [53], [30]
7	Smoking	[42]
8	Respiratory/pulmonary disease	[42], [48]
9	Ejection fraction	[42] [48], [49], [32], [34], [51], [35], [30], [52]
10	Renal function	[42], [33], [41]
11	Arrhythmia	[42]
12	Hypercholesterolemia	[42]
13	Prior cardiac surgery	[31]
14	Critical preoperative condition	[31]
15	Preoperative angina requiring intravenous nitrates	[31]
16	The use of the radial artery (RA) as a second conduit in nonobese patients	[47]
17	Second arterial conduit	[48]
18	Logistic European System for Cardiac Operative Risk Evaluation score (EuroSCORE)	[48], [43]
19	New York Heart Association (NYHA) Class	[48]
20	Previous myocardial infarction	[48], [51]
21	Extracardiac arteriopathy	[43]
22	Left main stem disease	[43]
23	Galectin‐3 levels	[37]
24	Serum telomerase enzyme level	[38]
25	Levels of 25(OH)D	[38]
26	BMI	[49], [33]
27	Opium consume	[49]
28	Chronic obstructive pulmonary disease (COPD)	[50]
29	Prolonged length of stay (PLOS)	[50]
30	Complete revascularization	[32]
31	Intraoperative mild hypothermia	[33]
32	Nasopharyngeal CPB (cardiopulmonary bypass) temperatures of 32°C and 33°C	[33]
33	Inflammatory markers	[33]
34	Complications	[34]
35	Female gender	[51]
36	Preadmission glycemic control	[40]
37	Left internal thoracic artery use	[53]
38	Surgeon experience	[35]
39	Ventricular tachycardia (VT)	[44]
40	Ventricular fibrillation (VF)	[44]
41	Serum creatinine	[30]
42	Type of medical intervention	[52]
43	Preoperative atrial fibrillation	[46]

### 3.7 The Distribution of Included Papers Based on Survival Rate

The survival rate of patients undergoing CABG was very variable, and 1‐, 5‐, 10‐, 15‐, and 20‐year survival rates were reported in some studies. Various factors, such as diabetes and other diseases, affected the survival rate, detailed in Tables 2 and 3.

### 3.8 The Limitation of Included Papers

The limitations and challenges mentioned in the studies are given in Table 4. Two studies did not mention any limitations or challenges. According to the table below, it is clear that different types of bias were the most important limitation of the reviewed studies.

**Table 4:** Limitation of reviewed studies.

#	Limitations	Studies
1	Selection/inherent/recall/sampling/data collection/residual bias	[31], [36], [41], [43], [45], [47], [50], [53], [51], [42], [32]
2	Retrospective nature of the study	[31], [32], [35], [39], [41], [48], [45], [53], [50], [43]
3	Single‐institutional/center	[35], [39], [41], [48], [36], [32], [33], [45]
4	Limited generalizability	[33], [34], [35], [45], [51], [44], [46]
5	Focusing only on CABG surgery not all cardiac operations	[33], [40], [45], [34]
6	Observational nature	[35], [36], [34], [47]
7	The presence of confounders	[31], [34], [35]
8	Non‐randomized/random assignment	[32], [41], [53]
9	Long study period	[41], [50], [35]
10	Lack of data about the cause of death	[42], [53], [47]
11	Only consider/rely on patients' characteristics and variables available before surgery, limiting the accuracy of predicting all‐cause mortality	[37], [42]
12	Does not include emergent patients	[38], [53]
13	Limited by a lack of data for major adverse cardiovascular events	[48], [32]
14	Considering creatinine as the be the best marker for renal function	[30]
15	Not considering South Asian ethnicity, which has diverse subgroups, leading to varying preoperative variables	[30]
16	Data set Inability to define stroke severity and its impact on patients	[31]
17	Lack of information about the proportion of stroke patients who underwent imaging	[31]
18	No investigation of intraoperative factors' association with perioperative stroke	[31]
19	Basing on a single surgeon, and single technical configuration operative experience	[32]
20	Missing some important preoperative characteristics	[32]
21	Evaluating only long‐term survival	[32]
22	Not including co‐morbidity and medication data due to being unreliable in completeness and collection	[33]
23	Not including data on detailed cardiac performance such as ejection fraction	[33]
24	Association between body temperature and mortality does not imply causality	[33]
25	Unknown influence of the surgical team regarding temperature management	[33]
26	Not providing information regarding the type of diagnostic study from which LVEF was ascertained and the timing of the study relative to surgery	[34]
27	Missing some data in Data set, like the degree of myocardial viability and information on how complications were managed	[34]
28	Measuring surgeon experience by years of practice	[35]
29	Lack of distinction between patients with acute coronary syndrome and chronic coronary syndrome, which might affect our post‐CABG survival results	[36]
30	The data used was from 2004 to 2007, when heart disease‐related deaths were slightly higher than current rates	[37]
31	No use of SYNTAX score for coronary lesions	[39]
32	Lack of data on internal mammary artery graft in the redo‐CABG group	[39]
33	Small cohorts due to decreased frequency of redo‐CABG	[39]
34	No follow‐up of blood glucose control after discharge from the hospital	[40]
35	Different surgeon's procedures on patients undergoing CABG	[41]
36	Deriving GFR by using the MDRD formula	[41]
37	Defining the level of AKI based on creatinine increase within 48 h may interfere with initial RIFLE criteria	[41]
38	Potential outdated mortality risks due to using data from patients operated on a decade ago	[42]
39	Not available data for secondary prevention and control of risk factors and compliance with medical therapy	[43]
40	Not assessing angiographic graft patency for its correlation to survival	[43]
41	Not allowing further analysis for confounders such as surgical technique due to small group numbers and limitation of data	[43]
42	Challenges in defining urgent CABG due to the limitation of the STS database and other factors	[45]
43	The negative troponin cohort may include a heterogeneous population as the urgent and emergent STS definitions can apply to a wide variety of indications, including high‐risk coronary lesions, unstable angina, or heart failure	[45]
44	Missing troponin level of the patient before transferring to the institution	[45]
45	Incomplete linking between the STS and CMS databases	[46]
46	Limitations to both registries (STS and CMS), such as lack of data for the type of preoperative AF, causality of AF, AF status post‐procedure, and anticoagulation status	[46]
47	Excluding some patients with AF who received surgical AF ablation during CABG	[46]
48	There is no measurement of changes in BMI during follow‐up, so causality between parameters cannot be determined	[47]
49	Obesity is defined only by BMI, not actual measure of adiposity	[47]
50	No available follow‐up data for recurrence of angina, repeated revascularization, and graft patency	[47]
51	Having a small number of cases in the propensity‐matched groups	[48]
52	Do not distinguish between cardiac and noncardiac deaths	[48]
53	Lacks functional testing and angiographic correlation between survival and graft patency	[48]
54	Not repeated pulmonary function tests before surgery	[50]
55	Effect of medical therapy potentially resulting in misclassification of COPD severity	[50]
56	Adjusting for corticosteroids and other medications may have led to over adjustment	[50]
57	The database does not collect socioeconomic position (due to some reason), education, and income, and these factors may have influenced survival	[50]
58	The association between COPD and poor survival may have noncausal or noncardiac causes	[50]
59	The study's classification of COPD should be interpreted in comparison with other staging systems	[50]
60	The STS classification system does not take into consideration extrapulmonary abnormalities such as obesity‐related hypoventilation syndrome, pulmonary hypertension, and deconditioning	[50]
61	Did not differentiate results based on insulin dependence and not insulin‐dependent diabetes	[51]
62	Didn't investigate the incidence of coronary re‐interventions (PCI, redo‐CABG), hospital readmissions, or bypass graft patency	[51]
63	Changing risk profile of the population due to treatment changes in the follow‐up period	[51]
64	Exclusion of patients with obesity or renal failure may alter study results	[53]

### 3.9 Risk of Bias Assessment

The results of the risk of bias assessment in the reviewed articles are shown in Table 5.

**Table 5:** Risk of bias summary regarding each risk of bias item for each included study.

	Data collection	Study response	Outcome measurement	Statistical analysis and reporting
Salmasi 2023	Low	Low	Low	Low
Jonsson 2022	Low	Unclear	Unclear	Low
Jegaden 2022	Unclear	Unclear	Low	Low
Hendriks 2022	Low	Unclear	Low	Low
omer 2022	Low	Low	Low	Low
Han 2021	Low	Low	Low	Low
Sattartabar 2021	Low	Unclear	Low	Low
Parker 2020	Low	Low	Unclear	Low
Zarei 2020	Low	High	Unclear	Low
Gallo 2020	Unclear	Low	Unclear	Low
Robich 2019	Low	Low	Low	Low
Wang 2019	Unclear	Unclear	Low	Low
Karim 2018	Low	Unclear	Low	Low
Luthra 2018	Unclear	Unclear	Low	Low
cheng 2018	Unclear	Low	Low	Low
Beller 2018	Unclear	Unclear	Low	Low
Malaisrie 2018	Low	Low	Low	Low
Benedetto 2017	Low	Low	Low	Low
luthra 2017	Unclear	Low	Low	Low
Najafi 2016	High	High	Low	Low
Efird 2016	Low	Low	Low	Low
Pang 2016	Low	Low	Low	Low
Farhadi 2016	High	High	Low	Low
Singh	High	Low	Low	High

## 4 Discussion

The importance of predicting patient survival rates and the factors affecting them is well recognized. Today, the survival rate of patients, even after emergency surgeries, is approximately over 90%. However, it remains essential for individuals to know how long they are likely to live after various illnesses or surgeries [54, 55]. The advancement of emerging technologies, such as artificial intelligence, has enabled the world to predict patient survival rates using machine learning [56].

This study was conducted to determine the factors affecting the survival rate of patients after CABG surgery. It reviewed all articles using machine learning methods to extract patient survival rates or the factors influencing them and met the authors' inclusion criteria.

The results of this study identified 43 factors affecting the survival rate of patients after CABG surgery, among which factors such as age, diabetes, stroke, ejection fraction, and kidney function appear to be of greater importance.

Unlike some studies conducted in this area, which only examined preoperative factors [54, 57], the results of this study led to the identification of factors affecting patient survival rates pre‐, intra, and postoperative, as well as some underlying factors. For example, among the identified factors are a history of cardiac surgery and critical conditions related to before the surgery, mild hypothermia and the use of the left internal thoracic artery during the surgery, and finally, the factor of prolonged hospitalization related to the patient's condition after the surgery.

The role of age as an influential factor in the survival of patients after CABG surgery needs no discussion; however, it is unclear why the role of age as an influential factor in the survival of patients after some surgeries is overlooked. For example, the study by Maldonado et al. [58], which examined the relationship between age and the survival of patients after gastric cancer surgery, showed no correlation between age and 5‐year survival of patients. Meanwhile, in the same year, the results of the study by Brown et al. indicated that there is a significant relationship between being over 70 years old and the survival of patients after gastrectomy following gastric cancer [59].

Among the 24 articles included in the final analysis for the systematic review in our study, diabetes was mentioned as an influencing factor on patient survival in four (approximately 16%). The role of diabetes as one of the main underlying factors affecting the outcomes of CABG surgery is clear, as the results of the study by Kusu‐Orkar et al. [60] showed that diabetes, along with factors such as age, can impact surgical outcomes. Of course, this issue is not limited to cardiac surgeries; diabetes can also affect patient survival in noncardiac surgeries. For instance, the study by Zhang et al. indicated that diabetes influences the length of hospital stay, the occurrence of postoperative complications, and mortality after noncardiac surgeries [61]. It seems that diabetes can hinder the wound‐healing process [62] and increase the risk of complications, such as infections [63] or acute kidney injury [64]. Additionally, many patients with CAD often have comorbidities like hypertension and obesity, which may also influence their survival rates [65].

Ejection fraction is essential in evaluating cardiac function and can help predict patient outcomes after surgery. A normal ejection fraction typically ranges from 50% to 70%, while an ejection fraction of less than 40% is classified as low. Research indicates that a low ejection fraction before surgery is linked to the incidence of postoperative complications like respiratory failure, pneumonia, stroke, sepsis, deep sternal wound infection, and bleeding. These complications can lead to various problems for patients. Based on this information, it becomes clear why ejection fraction is associated with patient survival [66].

A cerebrovascular accident, commonly known as a stroke, is considered one of the nonlife‐threatening complications associated with cardiac surgery. Patients who experience complications like a stroke after surgery typically require a longer postoperative hospital stay. This extended hospitalization can increase the risk of infections within the hospital environment, further impacting survival rates [67].

Renal function is significantly impaired following on‐pump heart surgery. Patients who experience renal dysfunction after CABG are at risk for various complications. Effective fluid management is crucial, as failure to address this can lead to issues such as pulmonary edema. Additionally, these patients face a higher likelihood of postoperative complications and delayed recovery, all of which highlight the impact of renal function on survival rates after CABG surgery [68, 69].

Given the complexity of physicians' predictions regarding survival and the influencing factors on the one hand and the extensive coverage of the extracted factors in our study regarding patient conditions on the other hand, it is hoped that relevant specialists can not only predict patient survival but also tailor their treatments based on these factors.

Additionally, the present study's results showed that the Cox regression method has been used more frequently in the included articles among machine learning methods for predicting survival rates and related factors. However, according to the results of the study by Almazrouei [70], which compared machine learning algorithms for predicting survival in cardiac patients, decision tree methods may perform better than methods such as logistic regression, artificial neural networks, and support vector machines in predicting survival rates. The results of a study by Li et al. [24] also nearly support this finding. Although the focus of the authors of this article was on the survival of patients after breast cancer, the results of this systematic review indicated that decision tree methods had more applications in the reviewed articles.

Even though this seems to be the first study to systematically extract factors affecting patient survival after CABG, this study also has limitations. One limitation is that the extracted factors only cover the survival rates of patients after CABG performed using the on‐pump method and cannot be generalized to all CABG surgeries like those performed using the off‐pump technique. Another limitation of this study was the time constraint imposed, such that studies published before 2015 were not included in the review according to the inclusion criteria. This was because the authors wanted to report the most recent results in patient survival and machine learning.

## 5 Conclusion

This study was conducted to identify the factors affecting the survival of patients after CABG using grafts. The results of this study showed that 43 factors influence the survival rate of patients, among which some factors, such as age, diabetes, stroke, ejection fraction, and kidney function, appear to be of greater importance. It is expected that the extracted factors, in addition to identifying high‐risk patients for the healthcare system, will assist specialists in taking effective measures to manage the conditions of these patients.

## Author Contributions

Behzad Imani, Soheila Saeedi, Amir Shams, and Alireza Jafarkhani developed the concept for the study. Soheila Saeedi and Alireza Jafarkhani screened the articles under the supervision of Behzad Imani. Soheila Saeedi and Alireza Jafarkhani conducted the analysis and interpretation under the supervision of Behzad Imani. Finally, the manuscript was drafted by Behzad Imani, Soheila Saeedi, Amir Shams, and Alireza Jafarkhani. All authors reviewed the content and approved it.

## Conflicts of Interest

The authors declare no conflicts of interest.

## Transparency Statement

The lead author, Behzad Imani, affirms that this manuscript is an honest, accurate, and transparent account of the study being reported, that no important aspects of the study have been omitted, and that any discrepancies from the study as planned (and if relevant, registered) have been explained.

## Data Availability

The data supporting the findings of this study are available within the article or its supplementary materials.
